# The Atlanta Medical Society

**Published:** 1882-12

**Authors:** 


### Societies.

### THE ATLANTA MEDICAL SOCIETY.

*Reported for the Atlanta Medical Register.*

ATLANTA, October 21, 1882.

The president, Dr. W. S. Armstrong, in the chair.

Dr. James B. Baird reported a case of fracture of one of the cornua of the os hyoides in the person of a gentleman who was violently seized at the throat by a powerful employee and forcibly borne to the ground. The throat not only sustained the violence of the muscular grasp but supported, after he was prostrated, a considerable part of the weight of his assailant. Certain movements of the tongue were painful, so was swallowing and efforts to ingest solid food were attended by special difficulty—indeed it was almost impossible to get down a bolus.

Quietude was enjoined and his diet was restricted to fluids for several weeks. The trouble gradually subsided.

Dr. Baird also reported the application of ether spray to the back of the neck in a case of severe and obstinate post-cervical neuralgia or "crick."

He had seen the suggestion recently in a medical journal [Dr. J. T. McColgan in the *Southern Practitioner*] and resorted to it somewhat doubtfully after failing to relieve his patient—a nervous and delicate lady—with quinine, counter irritation, fomentations, etc., with the most satisfactory results. Two applications, one in the morning and one in the evening of the same day, afforded prompt and permanent relief.

The spray was applied by means of an ordinary hand-ball atomizer, directly to the painful part, and continued until a completely blanched spot of two or three inches in diameter was produced.

The process was not painful and scarcely disagreeable, and the results were all that the most exacting could desire.

He has frequently used galvanism, in such cases, with success, but it occasionally disappoints his expectations. He did not employ it in the case reported on account of the inconvenience of making the application in that particular instance.

Dr. John G. Earnest liked the suggestion of the ether spray and had no doubt it would prove a valuable therapeutic resource in these cases. He thinks, however, that all cases of this kind can be relieved by the use of galvanism, but to insure success the strength of the current must be sufficient—sometimes of a strength of thirty or forty cells.

Dr. W. S. Elkin reported the following case of dysentery: A roofer, during the heat of summer, after working all day in the sun, ate imprudently, and developed a severe attack of acute dysentery. He had had eight to twelve actions, accompanied by tenesmus, and composed of blood and mucus, daily for several days. When first seen he was very feeble. Ordered a dose of castor oil, after the action of which gave twenty-five grains of ipecac, repeated three times in the course of eighteen hours. Each dose was preceded by twenty drops of laudanum. The patient had, of his own accord, taken occasional doses of laudanum since the commencement of the attack without appreciable benefit.

The ipecac should be given in as little water as possible to avoid exciting nausea. Every dose was followed by quiet sleep. The stools soon became normal, and recovery promptly ensued. Only one motion contained blood, and that in very small quantity, after the first dose of ipecac.

Dr. Earnest has often followed this plan of treatment. He formerly preceded the ipecac by a dose of opium, but has latterly discontinued the use of the opium in that way. He gives twenty-five or thirty grains of ipecac for the first dose and remains by the side of the patient to note the effect. If the first dose is retained the others are not likely to be rejected. Thinks even if the ipecac causes vomiting that it does good. Has been occasionally disappointed in its use. Recently encountered an extreme case of dysentery. The patient was almost narcotized from free use of opium, yet he continued to have involuntary evacuations. He resorted to hot water injections. The man went to sleep and recovered. Has since resorted to the same treatment. Twenty to twenty-five minutes, sometimes, are sufficient to demonstrate good results. The rectum at first resists the intrusion but it soon becomes quiescent. He sometimes uses the catheter by the side of the injecting tube to facilitate the return of the water.

Dr. W. S. Armstrong said he was called, a few days ago, to see an old negro woman who was standing in a chair washing a window, when she fell, thrusting her hand through the pane. In the descent she managed, with the aid of the broken glass, to sever the radial and interoseous artery in the fleshy part of the forearm. The hemorrhage was so free that she fainted and the flow ceased. When he arrived he found the circulation so depressed that he administered brandy. She revived but there was no return of the hemorrhage. If the bleeding had recurred he should have cut down upon the vessels and ligated the cut ends, but, after waiting three quarters of an hour and no return of the hemorrhage, he was compelled, by other engagements, to leave the patient. So he packed the wound with lint saturated with the tincture of the chloride of iron—the officinal muriated tincture—and applied a roller bandage. She had no trouble afterwards but pain in the wound. On the fifth day he removed the packing and the raw surfaces looked charred—the effect of the iron. He continued to dress with cotton and a salve of vaseline and bromo-chloralum. Now, two weeks and three days from the date of the accident, the wound is healing kindly and has progressed without any untoward symptom.

Dr. Earnest, in the discussion following the recital of this case, said under similar circumstances he thought he should wash out the wound with an antiseptic solution and apply a light dressing, with precautions against danger from sudden hemorrhage, in the hope that he might be able to find and tie the severed or wounded vessels when the hemorrhage recurred, after full reaction had taken place. But if he packed the wound as advocated and practiced by Dr. Armstrong, he prefers alum to the muriated tincture of iron. It may not be so prompt in its astringent action as the iron but it is a good disinfectant, and he thinks the resulting clot is not so firm and that it actually contracts the mouths of the vessels better. He uses a satuated, watery solution applied on cotton. For flooding, he injects this solution into the cavity of the uterus after fully and completely dilating the os. But really thinks in many instances that hot water is better than any thing else.

Dr. Earnest continuing reported the case of an old gentleman who applied to him with a tumor on the back, just below the scapula and to the left of the spine. It was evidently filled with fluid. His diagnosis was a cystic tumor. There was no heat or other evidence of inflammatory action. An exploratory trocar was introduced without at first obtaining any fluid. After a while a little pus was discovered, which proved the tumor to be a cold abscess. It was laid open by a free incision, and about three gills of pus evacuated. At the bottom of the cavity he found a mass of cheesy matter, altogether about the size of a hen's egg. He washed out the sore with a solution of carbolic acid, and applied an antiseptic dressing.

After questioning, the patient recalled the occurrence of a slightly inflamed spot at or near the location of the abscess several months before, as the result of a light blow upon the part, but he attached no importance to it, and it had passed out of mind.

